# Associations of Blood Pressure with Body Composition among Afro-Caribbean Children in Barbados

**DOI:** 10.1371/journal.pone.0121107

**Published:** 2015-03-27

**Authors:** Pamela S. Gaskin, Ryan V. Hall, Peter Chami, Margaret A. St. John, David A. Gaskin, Oarabile R. Molaodi, Seeromanie Harding

**Affiliations:** 1 Faculty of Medical Sciences, University of the West Indies, Cave Hill, Barbados; 2 The Warren Alpert Medical School of Brown University, Providence, Rhode Island, United States of America; 3 Faculty of Pure and Applied Sciences, University of the West Indies, Cave Hill, Barbados; 4 CSO/MRC Social and Public Health Sciences Unit, University of Glasgow, Glasgow, United Kingdom; Loyola University Chicago, UNITED STATES

## Abstract

Despite complex presentation of adult hypertension and a concomitant obesity epidemic, little is known about overweight in relation to blood pressure among Caribbean children. We examined blood pressure in relation to body size in a cross-sectional study of 573 Barbadian children aged 9–10 years (2010-2011).The United States normative blood pressure percentiles were used to identify children with high (≥ 95^th^ percentile) or high normal blood pressure (90^th^ – 95^th^ percentile). The World Health Organization body mass index cut-off points were used to assess weight status.

**Major findings:**

Thirty percent of children were overweight/obese. Percentage fat mass differed between girls (20.4%) and boys (17.72%) (*p*< 0.05). Mean systolic blood pressure among girls was 106.11 (95% CI 105.05, 107.17) mmHg and 105.23 (104.09, 106.38) for boys. The percentages with high or high-normal mean systolic blood pressurewere14.38% (10.47, 18.29) for girls and 8.08% (4.74, 11.41) for boys. Height and body mass index were independent correlates of systolic and diastolic blood pressure. Mean systolic blood pressure was related to lean mass but not fat mass, while diastolic blood pressure was associated with fat mass index and overweight.

**Principal conclusion:**

One third of 9-10 year old children in Barbados were overweight/obese and 12% had elevated mean systolic blood pressure. BP was related to body size. These findings signal potential adverse trends in weight gain and BP trends for children growing up in the context of a country that has recently undergone rapid economic transition.

## Introduction

Hypertension (HTN) is a major public health burden in the Caribbean, closely related to overweight and the level of economic development.[1] Periera’s review (2009) of prevalence of hypertension in developed and developing countries showed that the prevalence in Barbados was higher than other countries in the Caribbean and Latin American region, Africa and most of Europe, and was highest in the world for women.[2] Hypertension is a risk factor for stroke, coronary heart disease, heart failure and chronic renal failure and its global importance is recognized, particularly in the context of increasing obesity. Blood pressure in childhood predicts future blood pressure [3,4] and the worrying trends of paediatric overweight have led to a surge in studies examining its impact on BP in children in both developed and developing countries.[5–7] The Caribbean islands represent part of the African Diaspora at different stages of transition, and the complex picture of high prevalence of HTN, but not of coronary heart disease among adults of African origin, is well known.[8] The social and economic context in the Caribbean may influence the development of blood pressure in children. For example, family circumstances may modify the relationship between weight status and blood pressure via socio-economic disadvantage.[9] In the Caribbean between 22%- 44% of households are classified as single parent households, highest in Barbados (44%).[10,11] Children living in single parent households are likely to be more obese and socio-economically disadvantaged than those in two parent households.[12] Studies of overweight and of BP in Caribbean children are therefore of both aetiological and clinical significance.

This is the first known study to examine the association between blood pressure and body size among children in Barbados, a country that was classified as ‘middle income’ in the 1980s, but now as a ‘high income’ in a region that is undergoing rapid, if unequal, economic transition.

## Materials and Methods

All applicable institutional and governmental regulations concerning the ethical use of human volunteers were followed during this research. The study was approved by the Institutional Review Board of the University of the West Indies/Ministry of Health Barbados. Informed written consent was obtained from parents and assent from children. A cross sectional survey was conducted between September 2010 to April 2011 of 617 (342 girls, 275 boys) 9–10 year old children from 22 government primary schools in Barbados. A sample of 634 children was required to estimate the prevalence of overweight within 3% of the true value with 80% power, assuming a prevalence of 25%. This estimate is based on a prevalence of 27% overweight using the International Obesity Task Force (IOTF) BMI cut-off points among Barbadian children 10–16 years in 1999 [13] and 26% overweight among children aged 15–18 years from southern Brazil in 2001–2002.[14] Given that the prevalence of overweight generally increases with age, we expected the prevalence among children aged 9–10 years in Barbados to be slightly lower than that of older children, due to a worldwide trend of secular increases.[15]

There are 74 government primary schools in Barbados and approximately 85.7% of all primary school aged children (5–11 years) in Barbados attended government schools in 2010–2011. In government schools 97% of the pupils were identified as Afro Caribbean. This corresponds with the race profile of the general population; 92% Afro Caribbean, 2.7% White, 3.1% Mixed, 1.3% Indian, 0.4% Other.[16] Three single-stage cluster sampling was used. Fifteen (15) of the 74 schools were selected using probability proportionate to size (PPS) sampling. Estimates were “self-weighting” (the size of clusters did not enter into computations of proportions or associated standard errors). The desired sample size was 634. Forty-two 9–10 year old children (21 boys and 21 girls) were randomly targeted from a class list until the quota was met. If subjects refused to participate in the study, recruitment continued until a total of 42 students were enrolled in each cluster. Seven (7) of the 15 schools could not meet the quota, 5 of which had fewer children than 42 enrolled in the 9–10 age group. These 7 schools were paired with a school of comparable characteristics in the neighbourhood. All invited schools agreed to take part. The pupil response rate was 46.8% in schools where the quota was reached and 47% in the pair schools. The Ministry of Education provided the 2009 school population details required for the sampling procedures.

Our pilot study showed that 50% of children under aged 9 years were unable to answer the questions posed during the interview and 10–11 year olds were busy with Secondary School entrance examinations.[17] We therefore invited only children in class 3 (the grade 5 equivalent, 9–10 year olds) to join the study. All measurements were obtained by trained observers. Quality control included reliability checks of measurements by the supervisor prior to and during data collection. Inter-observer reliabilities for height were conducted prior to the start of the study (N = 32) and intra-class correlation coefficients were high (> 0.97). Scales were calibrated by use of a standard weight (3kg). The field work supervisor visited the schools to ensure that the measurement protocols, for anthropometry and blood pressure were followed.

### Body-composition

All measurements were conducted under semi-private conditions prior to lunch and at least 2 hours after the last meal. Children were measured in uniform, with shoes and socks removed. Height was measured using a stadiometer (Charder Co. Ltd., HM200P, USA), weight using a digital scale and foot to foot measurement of bio-impedance analysis (BIA) using the TanitaTBF-300 Body Composition Analyzer. Standing height and weight were measured to 0.1cm and 0.1kg, respectively, following standard procedures [18]. Height and BMI were converted to z-scores using the WHO growth reference and AnthroPlus program [19]. Two consecutive measurements were performed for each anthropometric measure. The mean was used in data analyses. We calculated total body water from the resistance measurements using an equation validated for children.[20] Fat free mass was then calculated.[20] Fat mass was obtained by subtracting fat free mass from total body weight. Fat free mass and fat mass were presented as fat free mass index (FFMI = fat free mass/height^2^) and fat mass index (FMI = fat mass/height^2^) standardised for height.[21]

### Blood pressure

After a rest period of at least 10 minutes, BP was measured using a validated Omron HEM-907XL monitor, twice measured on the right upper arm with a one minute interval. The appropriate cuff size was selected based on mid arm circumference.[22] The mean value was recorded of the systolic (SBP) and diastolic (DBP) blood pressure. The US normative BP percentiles were used to classify children as high (≥95^th^percentile) or high normal BP (90^th^ – 95^th^percentile)[22]. We combined high normal and high BP and this is referred to as ‘elevated BP’ hereafter. Pulse rate was also recorded.

Household type (lone or two parent) was used as a proxy measure of socioeconomic status (SES). To assess household type children were asked whether they lived in the home with both parents (mother and father), one parent (single) or with one parent and others such as grandmothers or aunts (extended family). We aggregated the single and extended families into one category.

### Statistical Analyses

Summary descriptive statistics were obtained for all variables and the Kolmogorov-Smirnov test applied to verify whether the underlying distributions conformed to normality. The t-test was used to compare differences between means and chi-squared test used to compare differences in proportions of the variables. Pearson’s correlation was then carried out to investigate the associations between age, anthropometric and the BP variables.

To take into account the clustering of children within schools, multilevel regression models with school fitted as a random effect were used. The effect of age in 6 month categories, height z-score, BMI z-score fat free mass index (FFMI) and fat mass index (FMI) on BP (mean of 2 readings) was examined using multilevel-linear regression models, and on elevated BP (v. normal) using logistic models. BMI z-score was used as continuous variable then as a categorical variable with normal weight (-1≤ z ≤+1 SD), overweight (> +1SD) or obese (≥ + 2SD) classes. Household type was introduced in models adjusted for age and anthropometry. The interactions between all body size measures and household type were tested but were not significant. Data were analyzed using STATA (version 11).

## Results

From the total sample of 617 children, 575 children had data on height, weight, BIA and BP. Two children were excluded because of extreme BP values. The analyses were based on 573 (313 girls, 260 boys) children. Exclusions did not vary significantly by sex or age. About half of the children were in a lone-parent household. Table 1 shows the anthropometric and BP profiles of the sample.

**Table 1:** Body size, blood pressure, socio-economic circumstances (means and 95% confidence intervals) among 9–10 year olds in Barbados, 2010–2011.

	Girls (n = 313)	Boys (n = 260)	All (n = 573)*
Mean age(years)	9.71 (9.67,9.75)	9.72 (9.67,9.76)	9.71 (9.68,9.74)
Height (cm)	141.52 (140.66,142.38)	139.27 (138.45,140.09)	140.50 (139.89,141.10)
Height z-score	0.74 (0.61,0.88)	0.47 (0.34,0.60)	0.62 (0.53,0.72)
Weight (kg)	37.20 (36.06,38.33)	35.40 (34.08,36.72)	36.38 (35.52,37.24)
Weight z-score	0.75 (0.58,0.91)	0.64 (0.45,0.83)	0.70 (0.57,0.82)
Body mass index (kg/m^2^)	18.36 (17.93,18.80)	18.02 (17.51,18.53)	18.21 (17.88,18.54)
Body mass index z-score	0.48 (0.33,0.64)	0.42 (0.22,0.62)	0.45 (0.33,0.58)
% Fat free mass	79.56 (78.36,80.77)	82.28 (80.84,83.73)	80.80 (79.87,81.74)
Fat free mass index(kg/m^2^)	14.30 (14.18,14.42)	14.42 (14.30,14.54)	14.35 (14.27,14.44)
% Fat mass	20.44 (19.23,21.64)	17.72 (16.27,19.16)	19.20 (18.26,20.13)
Fat mass index (kg/m^2^)	4.17 (3.83,4.51)	3.68 (3.26,4.10)	3.95 (3.68,4.21)
%Overweight or obese (≥ +1SD)**	33.87 (28.59,39.14)	26.15 (20.78,31.53)	30.37 (26.59,34.14)
Mean SBP^a^ (mmHg)	106.11 (105.05,107.17)	105.23 (104.09,106.38)	105.71 (104.93,106.49)
% Elevated^b^SBP	14.38 (10.47,18.29)	8.08 (4.74, 11.41)	11.52 (8.90, 14.14)
Mean DBP^c^(mmHg)	62.62 (61.66,63.58)	62.72 (61.65,63.80)	62.67 (61.95,63.38)
% Elevated DBP	7.99 (4.97,11.01)	6.92 (3.82, 10.03)	7.5 (5.34, 9.67)
% Lone in parent household	52.69 (46.58, 58.80)	58.22 (51.54,64.89)	55.18 (50.68,59.68)

*Models adjusted for gender

^a^systolic blood pressure, elevated

^b^ BP ≥ 90percentile,

^c^diastolic blood pressure.

** overweight/obese, ≥ +1SD WHO references

Compared to boys, girls tended to be taller, heavier had more fat mass but there were no sex differences in BP. Twelve percent of the children had elevated systolic BP. Thirty percent of children were overweight. Mean pulse rate was significantly higher among girls than boys 91.2 ± 12.7 and 87.1 ± 12.6 beats/min (*p* < 0.001) respectively and among overweight children 90.9 ± 13.1 vs. 88.6 ± 12.6 beats/min (*p* = 0.044). Pulse rate was significantly related to both SBP (β = 0.083 95% Confidence Interval: 0.004, 0.163) and DBP (β = 0.122, 0.048, 0.196) among girls but only to DBP (β = 0.134, 0.054, 0.214) among boys.

### Prevalence of blood pressure and obesity

Around 6.5% (95% Confidence Interval 3.5, 9.5) for boys and 8.3% (5.2, 11.3) for girls were identified as having high-normal BP (systolic or diastolic). In addition, 6.9% (3.8, 10.0) of boys, and 9.6% (6.3, 12.8) of girls had high BP (systolic or diastolic). Six monthly increases in age were associated with an increase in SBP of 5.4 mmHg (95% Confidence Interval: 5.4, 5.5) among boys and 5.5 (5.4, 5.5) among girls and an increase in DBP of 3.2 mmHg (3.2, 3.3) among both boys and girls. SBP increased with height (cm) at 0.76 mmHg (0.75, 0.76) for boys and 0.75 (0.74, 0.76) for girls and DBP 0.45(0.44, 0.46) for boys and 0.44 (0.44, 0.45) for girls.

### Associations of blood pressure with significant variables

At the simple level SBP and DBP were significantly correlated with each other and the anthropometric variables (Table 2). In the regression models, height and BMI were independently related to SBP and DBP among both boys and girls (Table 3). In models with fat mass measures, BMI was not included due to the high correlation. FMI and overweight were significantly associated with DBP but not SBP. FFMI was associated with both SBP and DBP among girls, but with only SBP among boys. Height remained significantly associated with BP in these models. Household type was not associated with BP in any of these models.

**Table 2:** Pearson Correlation Coefficients of Blood Pressure and Anthropometry (N = 573).

	Mean SBP^a^ (mmHg)	Mean DBP^b^ (mmHg)	Age (years	Height (cm)	Weight (kg)	Body mass index (kg/m^2^)	% Fat free mass
Mean DBP^b^ (mmHg)	.563**						
Age (years	.034	-.002					
Height (cm)	.266**	.173**	.186**				
Weight (kg)	.236**	.238**	.120**	.682**			
Body mass index (kg/m^2^)	.184**	.225**	.068	.399**	.937**		
% Fat free mass^Ϯ^	-.172**	-.226**	-.089*	-.416**	-.897**	-.944**	
% Fat mass^Ϯ^	.172**	.226**	.089*	.416**	.897**	.944**	-1.000**

^a^systolic blood pressure,

^b^diastolic blood pressure

*. Correlation is significant at the 0.05 level (2-tailed).

^Ϯ^N = 565

**Table 3:** The effect of body size on Blood Pressure by gender, in 9–10 year olds in Barbados 2010–2011.

Systolic blood pressure, adjusted for age and body size	Girls (n = 313)	Boys (n = 260)	All, adjusted for gender (n = 573)
Age	0.045 (-1.41, 1.50)	1.12 (-0.46, 2.70)	0.41 (-0.67, 1.49)
Height z (adjusted for age)	1.99 (1.14, 2.85)*	2.51 (1.49, 3.52)*	2.20 (1.54, 2.85)*
Body mass index z (adjusted for age, height z)	0.89 (0.09, 1.69)*	0.96 (0.24, 1.96)*	0.95 (0.40, 1.49)*
Overweight/obese (adjusted for age, height z)	-0.13 (-2.39, 2.13)	1.15 (-1.49, 3.80)	0.42 (-1.32, 2.15)
Fat mass index (adjusted for age, height z)	0.13 (-0.23, 0.48)	0.24 (-0.12, 0.59)	0.18 (-0.08, 0.43)
Fat free mass index (adjusted for age, height z)	1.05 (0.08, 2.02)*	1.46 (0.31, 2.61)*	1.26 (0.51, 2.01)*
**Diastolic blood pressure, adjusted for age and body size**			
Age	-0.41 (-1.72, 0.91)	0.48 (-0.99, 1.95)	-0.01 (-1.00, 0.98)
Height z (adjusted for age)	1.18 (0.40, 1.96)*	1.34 (0.37, 2.31)*	1.30 (0.68, 1.91)*
Body mass index z (adjusted for age, height z)	1.33 (0.61, 2.06)*	0.87 (0.18, 1.56)*	1.08 (0.58, 1.59)*
Overweight/obese (adjusted for age, height z)	2.89 (0.83, 4.94)*	2.34 (2.34, -0.18)	2.65 (1.04, 4.26)*
Fat mass index (adjusted for age, height z)	0.51 (0.19, 0.82)*	0.47 (0.14, 0.81)*	0.48 (0.25, 0.71)*
Fat free mass index (adjusted for age, height z)	1.12 (0.24, 2.00)*	0.87 (-0.24, 1.99)	1.03 (0.33, 1.72)*

* *p* < 0.05

## Discussion

This study provides the first population-based estimates of the magnitude, distribution, epidemiology and burden of overweight and elevated BP among children in Barbados. About one third of Barbadian children were overweight. Barbadian children had similar BMI's to the same aged Black Caribbean children in the UK [18.21 kg/m^2^ (17.88–18.54) vs. 18.99 kg/m^2^ (18.82–19.17)]. They were less fat [19.20% (18.26–20.13) vs. 29.49 (28.95–30.02)] and were shorter [140.50 cm (139.89–141.10) vs. 143.0 cm(142.6–143.3)].[23] The prevalence of childhood obesity appears to be lower in Jamaica (which is a middle income country), 11% in 2007 among 10–11 year olds.[24] A recent study in Suriname in South America, classified as a low income country, also showed lower levels of overweight/obesity for African origin (Creole and Maroon) children aged 12–17 years old.[25] Mean SBP and DBP, and the prevalence of elevated BP, however, appeared to be similar to the British children (SBP: 105.71(104.93–106.49) vs104.9 (104.3–105.4); DBP: 62.67 (61.95–63.38) vs. 63.2 (62.7–63.7).[26] The prevalence of elevated SBP was: boys 8.08% (4.74–11.41) vs. 12.8% (2.4–17.2); elevated DBP: 6.92% (3.82–10.03) vs. 7.6% (2.9–17.2); elevated SBP girls 14.38% (10.47–18.29) vs. 16.1% (3.2–21.1); elevated DBP: 7.99% (4.97–11.01) vs. 11.0% (1.5–15.20.[5] The percentage of children with elevated BP among Black Americans aged 8–12 years in NHANES 2006 [27] was lower (3.5%), but the recently published estimate for 8–17 year olds in NHANES 2008 is much higher (19%).[28]

In this group of Barbadian children, height and BMIz were independent correlates of SBP across both sexes. The association of the body composition measures with BP were inconsistent for SBP and DBP and also differed between males and females. The consistent association of FFMI and SBP is in keeping with a positive effect of lean tissue development on SBP during growth.[29] As with our study Daniels et al.[30] found that FMI was related to DBP but not to SBP. This is important given that overweight and obesity tend to track into adulthood and that among US adults isolated diastolic HTN is shown to be more closely related to obesity than is systolic HTN.[31] The pathophysiology of adiposity to raised DBP is not well understood but may be related to cardiac output stroke volume and peripheral resistance.[29] Pubertal stage, known to be positively associated with BMI was not measured in our study. At age 9–10 years about half of African origin girls can be expected to be in late puberty, compared with less than 20% of boys.[32] The generally more consistent relationships for females than males with the body composition measures may be related to sex differences in puberty.[33] The consistent association of body size measures with DBP among females could be suggestive of earlier sub-clinical disease in females.

BMI may not adequately capture body composition particularly in African origin populations, among whom more lean mass can be expected compared with European children.[34] The Jamaican 1999 study suggested that SBP was more related to the lean mass than fat mass but the model fit was poor compared with models that included BMI.[35] We re-ran the analyses on overweight and obesity, adjusting additionally for FFM and found that as with the Jamaica study, BMI was more strongly related to DBP than FFM. This is in keeping with the finding of a greater association of obesity with DBP than SBP among youth of African origin [31,36].

There was a significant positive association of pulse rate to both SBP and DBP among girls but only to DBP among boys. Similar to findings among Canadian adolescents, this suggests increased sympathetic activity related to disturbances in the autonomic function related to a hyperkinetic state. In obese children, insulin may play a role in increasing the sympathetic nervous system and may promote sodium retention in the kidney.[37] Blood pressure has been shown to increase with increases with age.[38] The lack of an age effect in our study is possibly due to the narrow age band of the sample.

### Limitations

As mentioned above, pubertal status, a significant correlate of BP and body size, was not measured. Black African origin children have been shown to have earlier menarche than European children [5,39] but how this affects Black-White differences in BP trajectories with age is unclear. The narrow age band of children in this study reduces the bias from lack of adjustment of pubertal status. This is consistent with the use of age defined reference data for both body size [40] and blood pressure.[3] FFM was calculated using an equation validated in a mainly White population.[20] We combined both high and high normal BP due to the small sample size, but this is justifiable given that both are associated with early target organ damage in children.[41] Household type (lone or two parent) used as a proxy measure of SES was not associated with body composition. Parental occupation, education or income would have been better measures. The exclusion of children from private schools would have lowered the proportion of children of high SES.

The response rate was 47%. Ninety nine percent (99%) of the children who returned consent forms had parental consent. The most common reason for non-participation was that children forgot to take consent forms to parents or that they forgot to bring written consent to school on the day of measurement. Only 2 written refusals of consent were received. The response rate reduces the reliability of the prevalence estimate. However, given the similarity in patterns of associations with other studies,[42] these estimates will be valuable to power future studies in the Caribbean. Childhood overweight is clearly a significant public health burden in Barbados. Paediatric hypertension, associated with obesity, is likely to add to the public health burden of Barbados and rest of the Caribbean region. Chronic disease prevention and monitoring initiatives for children should be considered.

## Conclusion

One third of 9–10yr old children in Barbados were overweight/obese, 12% had elevated SBP. DBP was related to overweight. Mean BP and the percentage with elevated BP are similar to levels for the same aged Black Caribbean children in the UK. The results of this study support both lean and fat mass as correlates of BP. These findings signal potential adverse trends in weight gain and BP trends for children growing up in the context of a country that has recently undergone rapid economic transition.

## Supporting Information

S1 Table Barbados Children's Health and Nutrition Study (BCHNS) Blood Pressure Dataset. (XLS)
